# Data and the associated R code used to estimate health and economic burden of neurocysticercosis in India

**DOI:** 10.1016/j.dib.2016.02.079

**Published:** 2016-03-09

**Authors:** B.B. Singh, M.S. Khatkar, J.P.S. Gill, N.K. Dhand

**Affiliations:** a School of Public Health & Zoonoses, Guru Angad Dev Veterinary and Animal Sciences University, Ludhiana, Punjab 141004, India; b Faculty of Veterinary Science, The University of Sydney, 425 Werombi Road, Camden, 2570 NSW, Australia

## Abstract

This article contains epidemiological, demographic and other data used for estimating health and economic burden of neurocysticercosis (NCC)-associated active epilepsy in India [1]. Most of the data are embedded in the R-code used for analyses so that the reader is able to replicate the results or adapt the code to their own data. However, data used to conduct sensitivity analyses to evaluate the effect of changing important input values such as prevalence and per capita income on health and economic impact of NCC in India are included in tables. Results from sensitivity analyses are also presented in tables and figures. The paper also includes three scenarios with different age weighting (k) and time discounting (r) values used to estimate health and economic burden of NCC in India. The data for the scenario without any age weighting and time discounting are presented in “Estimation of the health and economic burden of neurocysticercosis in India” [1].

**Specifications Table**

**Table:** 

Subject area	Economics
More specific subject area	Health economics, neurocysticercosis (NCC)
Type of data	Table, figures
How data was acquired	Survey and data analysis
Data format	Analyzed data
Experimental factors	The study population, demographic, epidemiologic, disease severity and data associated with production losses.
Experimental features	Data were analysed using R-statistical program (R statistical package version 3.2.2, R Development Core Team (2015), http://www.r-project.org)
Data source location	India
Data accessibility	Data is within this article

## Value of the data

• The data and the code can be adapted to estimate health and economic impact of NCC in other countries.
• The data demonstrate the importance of input variables such as prevalence, per capita income, proportion of people seeking medical attention and case fatality on changing the disability adjusted life years (DALY) for NCC in India.
• The data demonstrate the importance of input variables such as prevalence and per capita income on economic losses occurring due to NCC in India.

## 1 Data

**Fig. 1:**
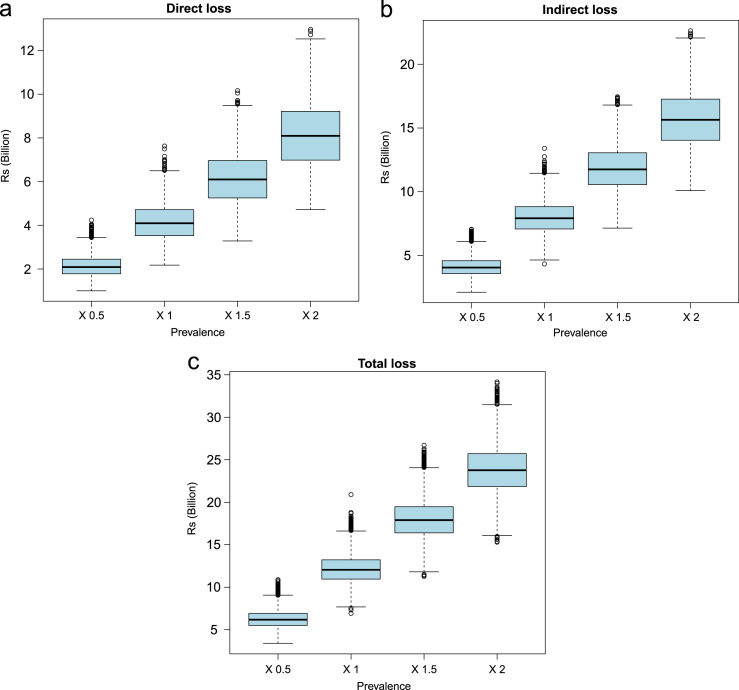
Sensitivity analysis to evaluate the effect of changing prevalence of neurocysticercosis (NCC) (0.5×, 1×, 1.5×and 2×(times) of the original input values) on economic losses associated with human NCC-associated active epilepsy.

**Fig. 2:**
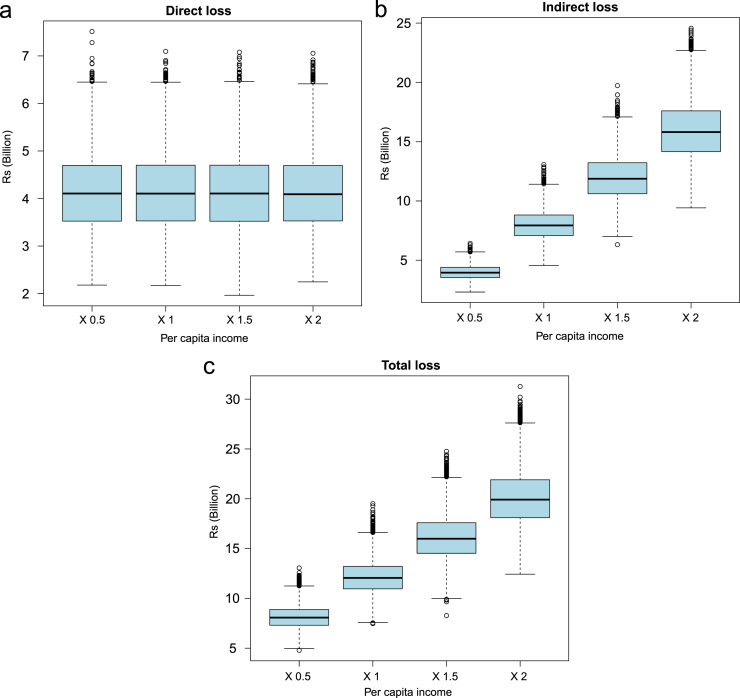
Sensitivity analysis to evaluate the effect of changing annual per capita income (0.5×, 1×, 1.5×and 2×(times) of the original input values) on economic losses due to human neurocysticercosis (NCC)-associated active epilepsy.

**Fig. 3:**
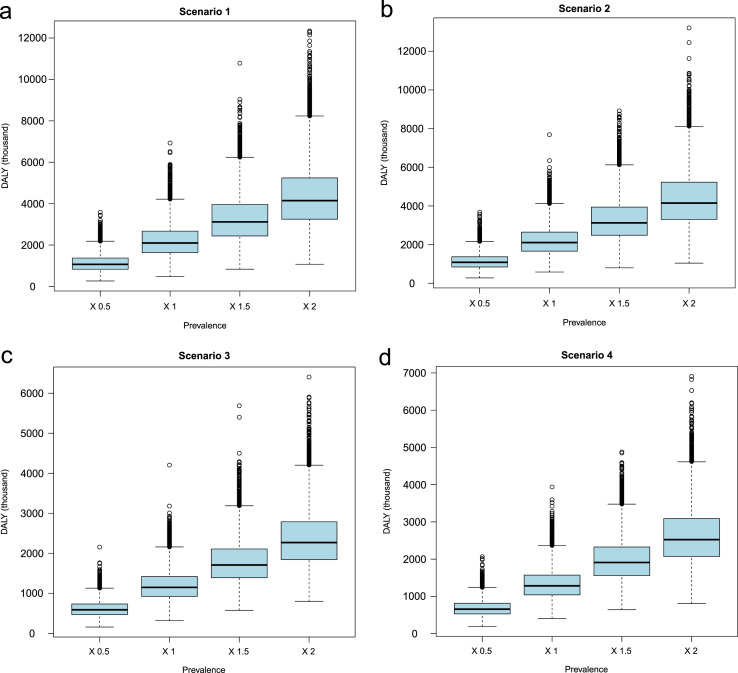
Sensitivity analysis of to evaluate the effect of changing prevalence of NCC (0.5×, 1×, 1.5×and 2×(times) of the input values) on disability adjusted life years (DALY) due to neurocysticercosis (NCC)-associated active epilepsy.

**Fig. 4:**
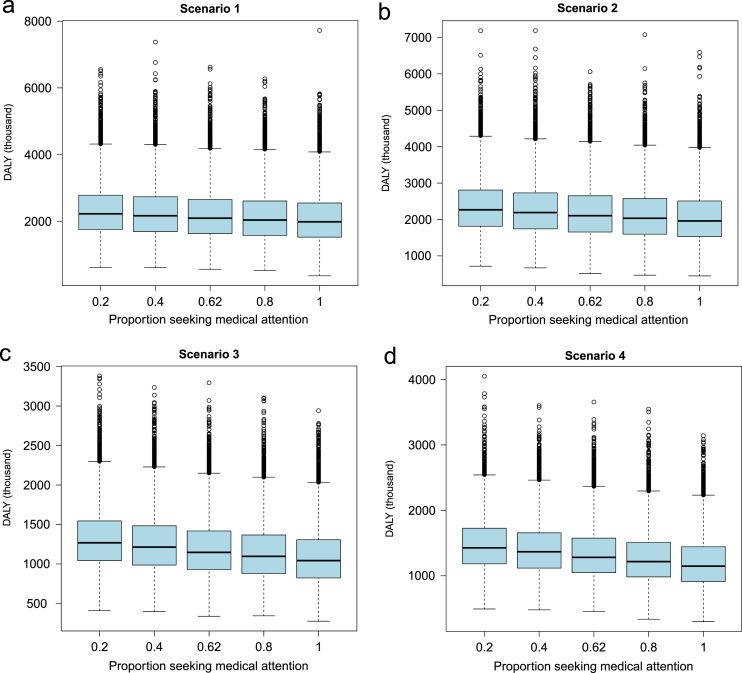
Sensitivity analysis to evaluate the effect of changing the proportion of human neurocysticercosis (NCC) cases seeking allopathic medical attention (from 0.2 to 1) on disability adjusted life years (DALY) due to NCC-associated active epilepsy.

**Fig. 5:**
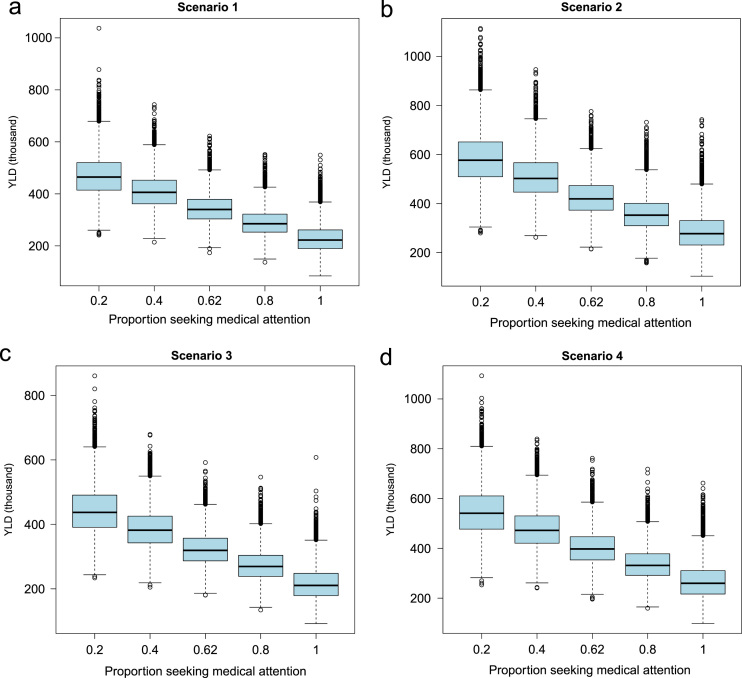
Sensitivity analysis to evaluate the effect of changing the proportion of human neurocysticercosis (NCC) cases seeking allopathic medical attention (from 0.2 to 1) on years of life lived with disability (YLD) due to human NCC-associated active epilepsy.

**Fig. 6:**
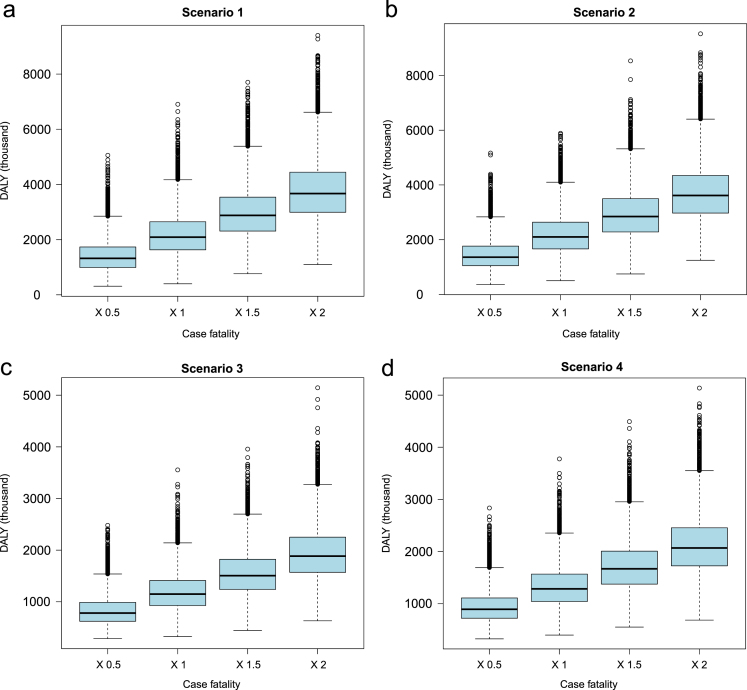
Sensitivity analysis of to evaluate the effect of changing case fatality of NCC (0.5×, 1×, 1.5×and 2×(times) of the input values) on disability adjusted life years (DALY) due to NCC-associated active epilepsy.

**Fig. 7:**
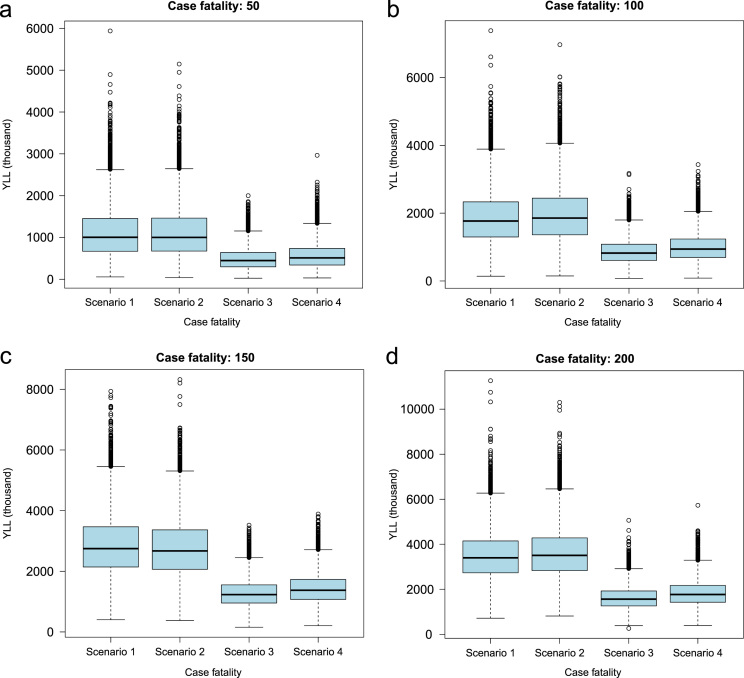
Sensitivity analysis to evaluate the effect of changing case fatality of neurocysticercosis (NCC) (0.5×, 1×, 1.5×and 2×(times) of the input values) on years of life lost (YLL) due to NCC-associated active epilepsy.

Demographic and epidemiologic data along with the associated R code used for estimating the health and economic impact are presented in R-code. The data indicating the effect of changing prevalence and per capita income on economic losses for NCC are presented in Figs. 1 and 2. The values of sensitivity analyses to evaluate the effect of changing prevalence, per capita income, proportion of people seeking medical attention and case fatality on DALY for NCC in India are presented in Table 1 and the associated results in Fig. 3, Fig. 4, Fig. 5, Fig. 6, Fig. 7. Results for three scenarios with different age weighting (k) and time discounting (r) are presented in Table 1 and the results for the scenario without any age weighting and time discounting are presented in “Estimation of the health and economic burden of neurocysticercosis in India” [1].

## 2 Experimental design, materials and methods

The demographic and epidemiologic data were collected by review of the national and international peer reviewed literature as well as sourced from government agencies [1]. The health and economic impact of neurocysticercosis was estimated as per published scientific literature [2], [3], [4]. The analyses were conducted (Fig. 1) in R-statistical programme (R statistical package version 3.2.2, R Development Core Team (2015), http://www.r-project.org). To compute 95% uncertainty interval (UI) (defined as 2.5–97.5th percentile), the authors ran Monte Carlo simulations for 10,000 iterations. The R code along with associated data [5], [6], [7], [8], [9], [10], [11], [12] has been provided as supplementary material (Appendix A).

## Figures and Tables

**Table 1:** Sensitivity analyses to evaluate the effect of changing prevalence, per capita income and proportion of people seeking medical attention on disability adjusted life years (DALY) for neuro-cysticercosis (NCC) in India. Results for three scenarios with different age weighting (*k*) and time discounting (r) are presented here; results for the scenario without any age weighting and time discounting are presented in Singh et al. [1].

*Parameters*	**Years of life lived with disability (in thousands)**		**Years of life lost (in thousands)**		**Disability adjusted life years (in thousands)**	
	**Median**	**95% UI**	**Median**	**95% UI**	**Median**	**95% UI**
**Scenario 2 (*k*=1; *r*=0)**						
**Prevalence**						
*Original input values***×***0.5*	216.29	93.66–469.26	861.4	114.6–3394.5	1081.7	276.8–3677.0
*Original input values*	421.5	220.7–794.6	1680.9	236.3–6992.9	2106	582–7688
*Original input values***×***1.5*	626.8	309.8–1159.8	2483.6	321.7–8012.6	3123.5	799.7–8918.3
*Original input values***×***2.0*	832.4	437.7–1468.1	3315.9	475.8–12092.4	4150	1040–13210

**Proportion of people seeking allopathic medical attention**						
*20% Patients*	577.0	280.0–1114.6	1682.2	239.1–6439.7	2265.6	717.4–7185.1
*40% Patients*	502.5	262.5–946.9	1676.6	232.9–6671.4	2189.6	671.1–7188.8
*62% Patients*	419.3	214.6–776.2	1675	226–5628	2104.0	512.9–6059.2
*80% Patients*	352.7	157.9–731.9	1671.1	203.1–6625.1	2033.0	470.1–7076.2
*100% Patients*	277.3	103.8–743.0	1672.0	232.1–6035.7	1959.5	450.6–6591.1
						
**Case fatality**						
*Original input values***×***0.5*	422.1	229.8–851.5	933.81	60.36–4661.44	1358.5	355.5–5166.3
*Original input values*	420.7	214.4–830.0	1671.5	166.3–5437.2	2098.4	500.1–5889.9
*Original input values***×***1.5*	419.7	202.3–764.7	2418.5	441.9–8124.8	2844.1	745.8–8536.1
*Original input values***×***2.0*	422.8	220.3–812.7	3184.5	810.4–8987.7	3614	1243–9526
				
**Scenario 3 (k=0; r=0.03)**						
**Prevalence**						
*Original input values***×***0.5*	164.68	79.89–335.99	420.13	54.29–1898.06	588.3	158.6–2156.4
*Original input values*	320.7	175.7–574.6	824.4	115.2–3789.5	1149.1	319.5–4205.2
*Original input values***×***1.5*	475.8	265.5–843.9	1230.4	177.0–4934.5	1708.0	575.6–5687.1
*Original input values***×***2.0*	630.8	376.2–1197.3	1631.7	245.5–5795.6	2268.8	799.3–6406.6

**Proportion of people seeking allopathic medical attention**						
20% patients	437.2	233.8–860.9	824.4	113.2–2944.1	1267.8	409.7–3382.6
*40% patients*	381.8	205.1–679.2	825.8	112.0–2800.3	1213.4	398.4–3235.9
*62% patients*	319.2	180.5–592.0	823.2	114.7–2770.6	1146.6	336.8–3294.5
*80% patients*	269.3	134.4–546.8	824.0	124.3–2862.2	1096.4	343.5–3101.9
100% patients	210.51	92.18–607.88	825.2	116.6–2684.8	1042.6	275.4–2941.8
						
**Case fatality**						
*Original input values***×***0.5*	320.4	158.4–559.6	457.40	29.59–2115.29	782.2	287.2–2481.6
*Original input values*	320.5	184.6–583.1	824.81	83.78–3206.29	1149.5	326.0–3554.6
*Original input values***×***1.5*	319.6	178.8–567.8	1185.0	196.2–3680.0	1505.8	445.5–3957.1
*Original input values***×***2.0*	320.8	172.9–664.6	1564.9	384.8–4791.1	1883.9	632.3–5144.9
				
**Scenario 4 (*k*=1; *r*=0.03)**						
**Prevalence**						
*Original input values***×***0.5*	203.54	88.35–430.85	448.94	51.49–1765.48	655.3	192.5–2065.8
*Original input values*	395.8	211.0–768.8	880.5	131.3–3470.5	1282.9	403.6–3938.6
*Original input values***×***1.5*	589.0	313.7–1025.3	1308.3	190.8–4298.8	1909.7	641.8–4878.9
*Original input values***×***2.0*	782.6	405.6–1310.6	1729.0	258.3–5974.9	2523.6	808.7–6907.9

**Proportion of people seeking allopathic medical attention**						
*20% Patients*	541.0	253.3–1093.2	877.3	116.2–3310.5	1426.4	491.8–4050.6
*40% Patients*	472.2	241.1–839.5	885.4	125.3–3112.1	1366.6	478.5–3601.7
*62% Patients*	397.4	196.1–762.0	878.6	107.2–3307.2	1281.5	455.4–3656.5
*80% Patients*	331.5	159.6–718.4	877.5	120.4–3101.9	1216.4	335.6–3548.5
*100% patients*	259.65	98.31–662.27	878.0	123.7–2894.9	1146.6	299.8–3142.2
						
**Case fatality**						
*Original input values***×***0.5*	397.3	211.2–760.6	489.03	27.44–2199.97	892.0	324.3–2835.4
*Original input values*	396.4	209.7–779.2	878.76	85.16–3249.93	1284	397–3777
*Original input values***×***1.5*	395.4	184.8–800.2	1265.6	238.2–4062.4	1668.7	550.6–4493.1
*Original input values***×***2.0*	396.8	201.1–727.3	1667.5	401.3–4647.1	2068.6	684.1–5136.5

95% UI=95% uncertainty interval (2.5–97.5th percentile)

## Footnotes

Appendix A Supplementary data associated with this article can be found in the online version at http://dx.doi.org/10.1016/j.dib.2016.02.079.
