# Supplementary material for: Data and the associated R code used to estimate health and economic burden of neurocysticercosis in India
Source: Data Brief. 2016 Mar 9;7:571–81. doi: 10.1016/j.dib.2016.02.079 (PMC4802432; doi:10.1016/j.dib.2016.02.079)
Supplement: Supplementary file 1 — Supplementary material [file mmc1.doc]

**Conflict of interest:** None
